# Induction of apoptosis and cell cycle arrest in colorectal cancer cells by novel anticancer metabolites of *Streptomyces* sp. 801

**DOI:** 10.1186/s12935-022-02656-1

**Published:** 2022-07-26

**Authors:** Arghavan Kouroshnia, Sirous Zeinali, Shiva Irani, Akram Sadeghi

**Affiliations:** 1 Department of Biology, Science and Research branch, Islamic Azad University, Tehran, Iran; 2 Department of Molecular Medicine, Biotechnology Research Center, Pasteur Institute of Iran, Tehran, Iran; 3 Department of Microbial Biotechnology, Agricultural Biotechnology Research Institute of Iran (ABRII), Agricultural Research, Education and Extension Organization (AREEO), Karaj, Iran

**Keywords:** Colorectal cancer, Actinomycetes, *Streptomyces*, Apoptosis, Cell cycle, LC–MS

## Abstract

**Background:**

Colorectal cancer is the third and most significant cause of death and fourth most common cancer in the world. Chemotherapy can be introduced in the cases of locally or distantly invasive colorectal cancer. In recent years Actinomycetes, especially the genus *Streptomyces*, contain numerous bioactive compounds, some of which are known as important anti-tumor chemotherapy drugs. In this research, we aimed to explore the anti-cancer mode of action of *Streptomyces* sp. 801 on colorectal cancer cells in vitro conditions.

**Methods:**

Fermented supernatant of strain *Streptomyces* sp. 801 isolated from soil showed maximum growth inhibition on human colorectal cancer cells. The cytotoxic effects of various concentrations of EtOAc extract from bacterial culture supernatant on HT-29, HCT 116 and SW480 cancer cells were surveyed using the MTT assay. Moreover, flow cytometry assays and *Bax*, *Bcl-2*, *Cyclin D1* and *P21* gene expressions were carried out to assess the apoptotic and cell cycle effects. Also, the scratch assay was performed to measure migration. Finally, Ethyl acetate (EtOAc) extract was analyzed by LC–MS to identify anti-cancer compounds.

**Results:**

The cell viability of all three cell lines were decreased in a dose-dependent manner. The successful induction of apoptosis and cell cycle arrest at IC_50_ values, were confirmed by flow cytometry as well as by the mRNA expression levels of the genes involved in these processes. Scratch assays indicated the inhibition of cell migration in the cancer cell lines treated by *Streptomyces* sp. 801. Nine anti-cancer compounds of *Streptomyces* sp. 801 were detected by liquid chromatography–mass spectrometry (LC–MS) analysis.

**Conclusions:**

These findings suggest that *Streptomyces* sp. 801 can be a source of promising anticancer metabolites.

**Graphical Abstract:**

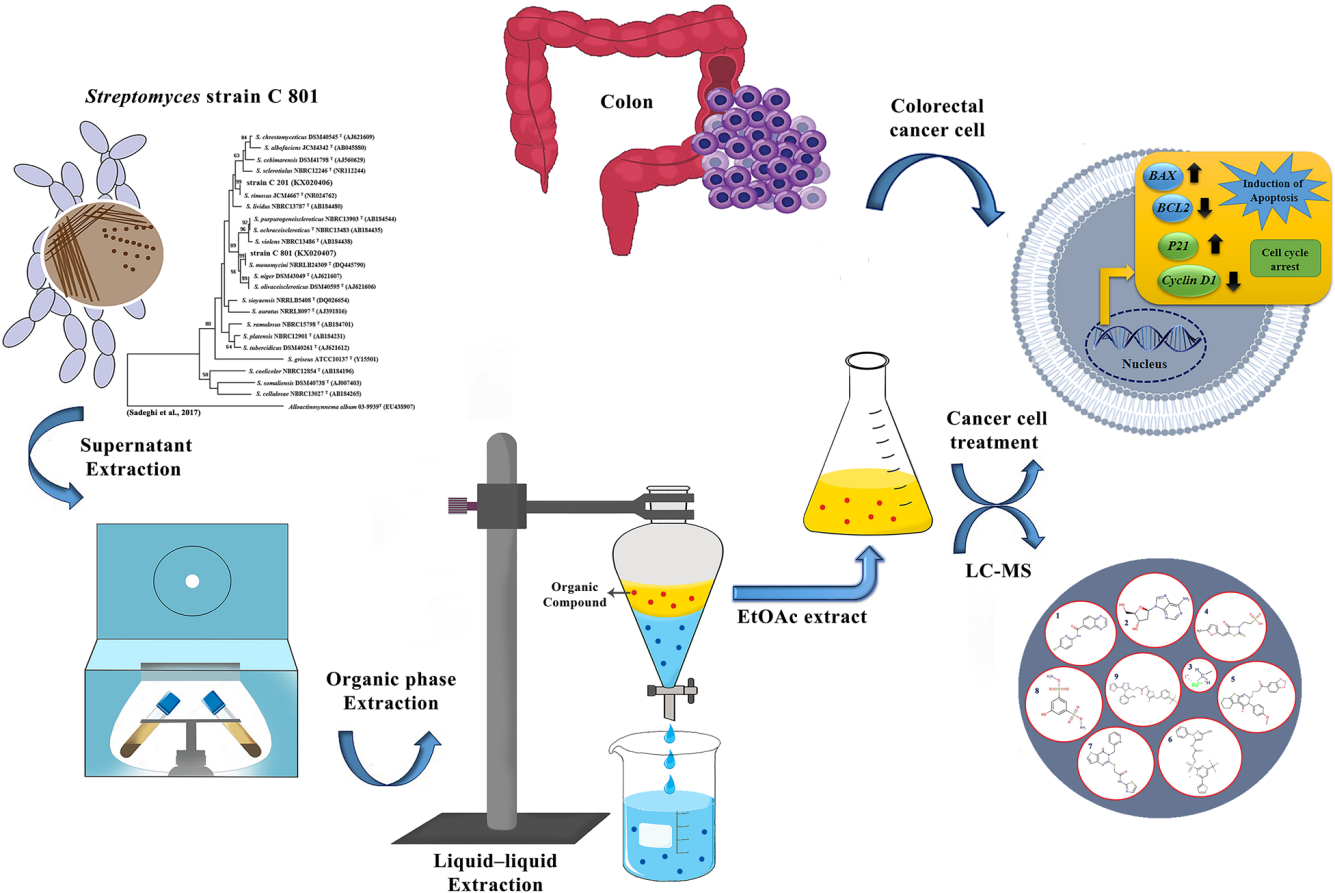

## Introduction

Nowadays, cancer is one of the major diseases that has affected human health. Colon cancer or colorectal cancer (CRC) is a serious malignancy with high incidence and mortality rates in both developed and developing countries [1]. The most common Gastrointestinal (GI) cancer is CRC, which encompass two common types of cancers, colon and rectal [2]. CRCs present the third most commonly diagnosed form of cancer worldwide, accounting for 11% of all diagnosed cancer cases 38.8% of GI cancer cases and 26.0% of GI cancer-related deaths [3–5]. Globally, CRC is the second most lethal cancer [6], an estimated nearly 900 thousand deaths per year at 2020 in both men and women [7] that causes widespread burden to healthcare system [8, 9].

CRC was considered a disease of adults aged 50 years old and above [10], however, prevalence among individuals younger than 50 years of age has been increasing during recent years by 2% per year [11, 12]. Though specific drugs have been approved to prevent malignancies and reduce mortality rates of CRC [13], still long-term side effects complicate the treatment and recovery of patients [14, 15]. Therefore, presenting drug alternatives derived from natural sources can be promising options cancer therapy. Numerous drug discovery endeavors focusing on natural products. To illustrate the significance of these studies, out of a total number of 1394 small-molecule drugs receiving international approval from 1981 to 2019, 67% were derived to natural products [16].

*Actinomycetes* are Gram-positive bacteria that have gained unprecedented relevance due to their various biological activities. These bacteria can be isolated from different ecosystems including soil, water, and marine sediments [17]. Most Actinomycetes are known to have biological and biotechnological significance. This phylum of gram negative bacteria has great potential to produce bioactive metabolites such as antibiotics, enzymes, herbicides, and anti-cancer compounds [18–20]. More than half of these compounds have been isolated from members of the genus *Streptomyces*. Many of these secondary metabolites are effective anti-tumor drugs. Anthracyclines (aclarubicin, daunomycin, and doxorubicin), peptides (bleomycin and actinomycin D), aureolic acids (mithramycin), enediynes (neocarzinostatin), anti-metabolites (pentostatin), carzinophilin, and mitomycins are examples of anti-tumor drugs with *Streptomyces* secondary metabolites [21, 22]. Accordingly, in recent decades, *Streptomyces* have received considerable research attention [23]. Some studies have confirmed the anti-cancer activities of *Streptomyces*-isolated compounds in both in vitro and in vivo experiments [24–27]. However, the mechanisms underlying their anti-cancer effects are not yet fully understood; thus, in-depth research on Actinomycetes is required.

*Streptomyces*, as mentioned, contain anti-cancer compounds that can be used as chemotherapy drugs [28] that is often used treat to control advanced cancers to enhance survival [29]. Among these cancers that usually spread to other organs, chemotherapy is the most common treatment for CRC [30, 31] that many lifestyle factors, obesity and dietary patterns are linked to the cancer [32, 33].

Here, the cytotoxic, apoptotic, and anti-metastatic effects of the organic phase of fermented supernatant of *Streptomyces* strain C 801 were evaluated against colorectal cancer cell lines to determine the mode of action of metabolites present.

## Materials and methods

### Bacterial source and culture conditions

**Fig. 1:**
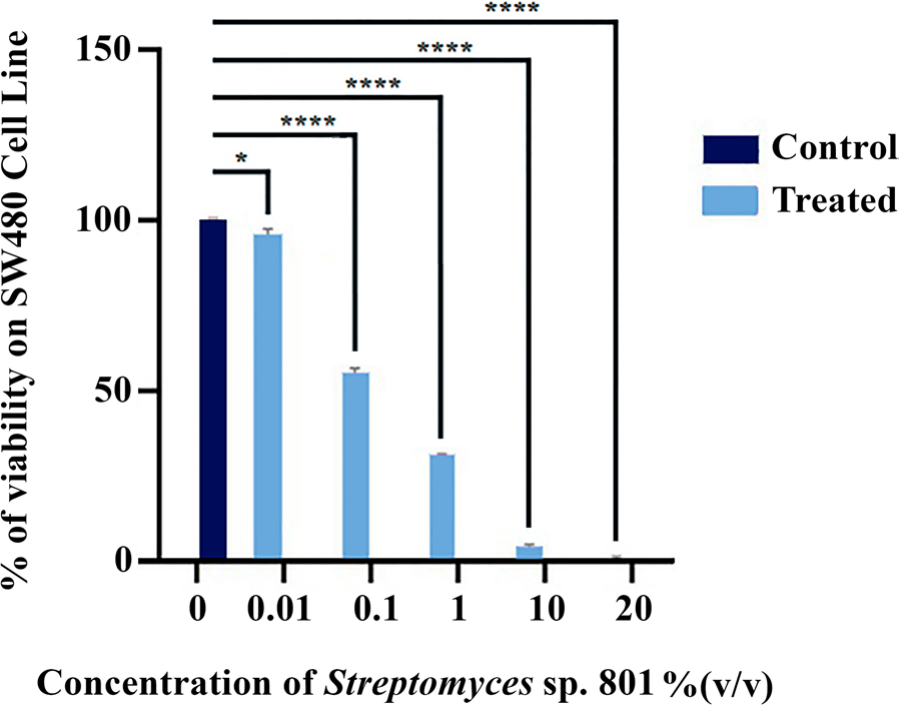
Inhibition activity of supernatant of *Streptomyces* sp. 801 against SW480 cell line at 2.04 (v/v) concentration

A bacterial culture collection containing 19 *Streptomyces* strains previously isolated from the rhizosphere of cucumber was used in this study. All strains showed more than 30% inhibitory effect against *Phytophthora drechsleri* causing cucumber damping-off. Strain C 801 with the highest biocontrol activity against the plant pathogen in the in vitro (78% inhibitory effect in dual culture test) and in vivo (reduced plant disease by 80% in greenhouse conditions) assays [34] showed the minimum proliferation and viability of human colorectal cancer cells, SW480 line (IC_50_ values of 0.1% v/v in the MTT assay) in an initial screening process using fermented bacterial supernatant (Fig. 1) (data for other 18 strains was not shown). Isolate C 801 (KX020407) was preserved and deposited in the Agricultural Biotechnology Research Institute of Iran Culture collection (ABRIICC). A single cell colony was streaked on the surface of an 8 cm plastic Petri plate containing “International *Streptomyces* Project media 2” (ISP2) medium (Kampfer et al., 1991) containing 10 g/L malt extract, 4 g/L yeast extract, 4 g/L glucose, and 18 g/L agar, adjusted to pH 7.2. The plates were incubated at 29 °C for 7 days. *Streptomyce*s spores were scraped off of the surface of the medium with a sterile spatula, suspended in sterile saline solution (0.9% NaCl), and adjusted to a concentration of 10^6^ CFU/mL. Spore suspension (100 µL) was added in Erlenmeyer flasks (250 mL) containing 50 mL broth ISP2 and incubated at 29 °C and 150 rpm in a rotary shaker incubator. After 4 days, the bacterial culture was centrifuged at 12,000 rpm for 5 min, and the supernatant was used for direct cell treatment or preparation of the organic phase.

### Organic phase preparation and LC–MS analysis

All reagents were purchased from commercial sources and used without further purification unless otherwise stated. All solvents used for compound purification were HPLC grade. The residue of cells and mycelia were removed from the bacterial culture supernatant via suction filtration through Whatman No. 5 filter paper, followed by 0.22 μm Millipore Durapore membrane filters. The crude mixture was dissolved in 250 mL of H2O and extracted with EtOAc (3 × 250 mL). The EtOAc fractions were dried and weighed. The EtOAc extract was dissolved in minimal H_2_O (10 mg/mL) and was used for cell treatments and LC–MS analysis (Waters Alliance 2695 HPLC-Micromass Quattro micro API Mass Spectrometer) [35, 36]. The spectra of biological fractions were analyzed and compared with the available library data and MZmine software. The LC–MS raw data files were converted to .mzXML format using MSConvert software to process mass spectral data for search in the spectral library and identification of known compounds. According to the literature, the data should first be aligned and normalized before the analysis step [37, 38].

### Cell culture

Three human colorectal cancer cell lines, SW480 (adenocarcinoma), HT-29 (adenocarcinoma), and HCT 116 (carcinoma) were obtained from ATCC. The cells were grown in RPMI-1640 medium (Gibco, Thermo Fisher Scientific, MA, USA), supplemented with 10% fetal bovine serum (FBS, Gibco, Thermo Fisher Scientific, MA, USA), and 100 U/mL penicillin and 100 µg/mL streptomycin (Gibco; Thermo Fisher Scientific, MA, USA) under a humidified atmosphere with 5% CO_2_ at 37 °C.

### MTT assay

Cell suspensions containing 1 × 10^4^ cells were plated in 96-well plates and incubated for 48 h to reach 80% confluency. After that, the culture medium was replaced with RPMI-1640 containing 100, 200, 400, 600, 800, 1000, 2000, and 4000 µg/mL of the EtOAc extract and incubated for 24, 48, and 72 h for all three cell lines. The cell viability was performed using an MTT assay. After this incubation time, 5 µg/mL of MTT solution (3, 4, 5-dimethylthiazol-2yl)-2, 5-diphenyltetrazo liumbromide; Sigma, Germany) was added to each well and incubated for 4 h. Then, the supernatant was replaced by 100 µL dimethyl sulphoxide (DMSO; Sigma-Aldrich, USA) and shaked for 15 min to dissolve formazan crystals that formed. Absorbance at 570 nm was then determined using a microplate reader (Bio-Rad Laboratories, Inc.). For the initial screening, the experiment was performed only on SW480 cells, and 0.01, 0.1, 1, 10, and 20 (v/v) concentrations of culture supernatant were added to each well and incubated for 48 h.

### Cell cycle analysis

SW480, HCT-116, or HT-29 cell lines at a density of 1 × 10^6^ cells/well were cultured in 6-well plates for 48 h and treated with 861.1, 293.6, and 29.8 µg/mL EtOAc extract, respectively, or with 0.1% DMSO as a control, for 48 h. The treated cells were detached using trypsin-EDTA, washed with 1X cold phosphate-buffered saline (PBS), and fixed in 70% ice-cold ethanol for 30 min at 4 °C. Prior to analysis, cells were washed with 1X PBS, centrifuged at 1800 rpm for 8 min, and incubated with 1 mL solution containing 100 µg/mL RNase A plus 50 µg/mL propidium iodide (PI, BD Biosciences, San Jose, CA, USA) for 1 h. Flow cytometry analysis was performed using BD FACSCalibur™ Flow Cytometer (BD Biosciences, USA). The accumulation of cells in cell cycle phases was determined using FlowJo software (v10.8.1, USA).

### Detection of apoptosis

The cells (1 × 10^6^ cells/well) were seeded in a 6-well plate and treated with different concentrations of EtOAc extract for 48 h. After the treatment period, the cells were detached, centrifuged at 3000 rpm, and suspended in 500 µL of 1X binding buffer. The cells were stained with 5 µL of Annexin V-FITC and 5 µL of PI for 5 min at room temperature in a dark situation, according to the manufacturer’s instructions (Apoptosis Detection Kit FITC; Invitrogen, USA). Untreated cells were considered as the control group. Quantification of apoptosis induced by the EtOAc extract was analyzed by flow cytometry using a BD FACSCalibur™ Flow Cytometer (BD Becton-Dickinson), and the acquisition data were interpreted using FlowJo software (v10.8.1, USA).

### Quantitative reversed transcription polymerase chain reaction (qRT-PCR)

Total RNA was isolated from both treated and untreated (control) cells using TRIzol solution (TRI Reagent®; Invitrogen, USA) according to related instructions. The integrity of RNA was assessed by agarose gel electrophoresis. According to the manufacturer’s protocol, the synthesis of first-strand cDNA was carried out using a RevertAid H Minus First Strand cDNA Synthesis kit (Thermo Scientific, USA). The qRT-PCR reactions were performed by SYBR Green Master Mix (TaKaRa Bio Inc.) and Applied Biosystems Step One PlusTM Real-Time PCR system (Thermo Fisher Scientific, USA). The primers for *Cyclin D1*, *P21*, *Bax*, *Bcl-2*, and *GAPDH* genes were designed and confirmed using AlleleID® software. The results were presented as the fold change in the target gene expression normalized to the glyceraldehyde 3 phosphate dehydrogenase (*GAPDH*) gene as the reference gene and relative to the control group. The details of the primers used are listed in Table 1.

**Table 1:** Primers used for quantitative RT-PCR

Target gene		Primer sequence (5′ to 3′)	Product length (bp)
*Cyclin D1*	Forward	CCCTCGGTGTCCTACTTCAAATG	109
	Reverse	CCTCCTCGCACTTCTGTTCC	
*P21*	Forward	GCTTCATGCCAGCTACTTCC	173
	Reverse	CCCTTCAAAGTGCCATCTGT	
*Bax*	Forward	ATGTTTTCTGACGGCAACTTC	133
	Reverse	AGTCCAATGTCCAGCCCAT	
*Bcl-2*	Forward	ATGTGTGTGGAGACCGTCAA	141
	Reverse	GCCGTACAGTTCCACAAAGG	
*GAPDH*	Forward	AACTTTGGCATTGTGGAAGG	132
	Reverse	GGATGCAGGGATGATGTTCT	

### Scratch wound healing assay

In vitro wound assay for evaluation of cell invasion was performed using SW480, HCT-116, and HT-29 cells in cultures that contained the EtOAc extract or vehicle control. Briefly, 1 × 10^6^ cells/well was seeded in 24-well culture plates overnight. A 1-mm wide linear wound was created across the center of each well with a pipette tip. Wounded monolayers were then washed once with PBS to remove cell debris and incubated in media containing EtOAc extract concentrations. For each well, five areas along the length of the wound were chosen randomly for photography under phase-contrast microscopy. After photography, the cells were incubated at 37 °C in a humidified incubator containing 5% CO_2_ and allowed to migrate for 48 h. Finally, the images were analyzed using ImageJ software.

### Statistical analysis

Statistical analysis was performed using GraphPad Prism (GraphPad Software, Inc., CA, US). Differences between groups were examined for significance with ANOVA and/or the t-test where appropriate and expressed as mean ± standard error of the mean (SEM). A value of *P < 0.05* was considered significant. All experiments were performed at least three times independently.

## Results

### Inhibitory effect of the fermented bacterial supernatant against cancer cells

First, the cytotoxic effects of the *Streptomyces* sp. 801 supernatant at 0.01, 0.1, 1, 10 and 20 (v/v) concentrations were tested to determine its anti-cancer effects on SW480 colorectal cancer cell after 48 h (Fig. 1).

### Isolation and selection of secondary metabolites of *Streptomyces* sp. 801

**Fig. 2:**
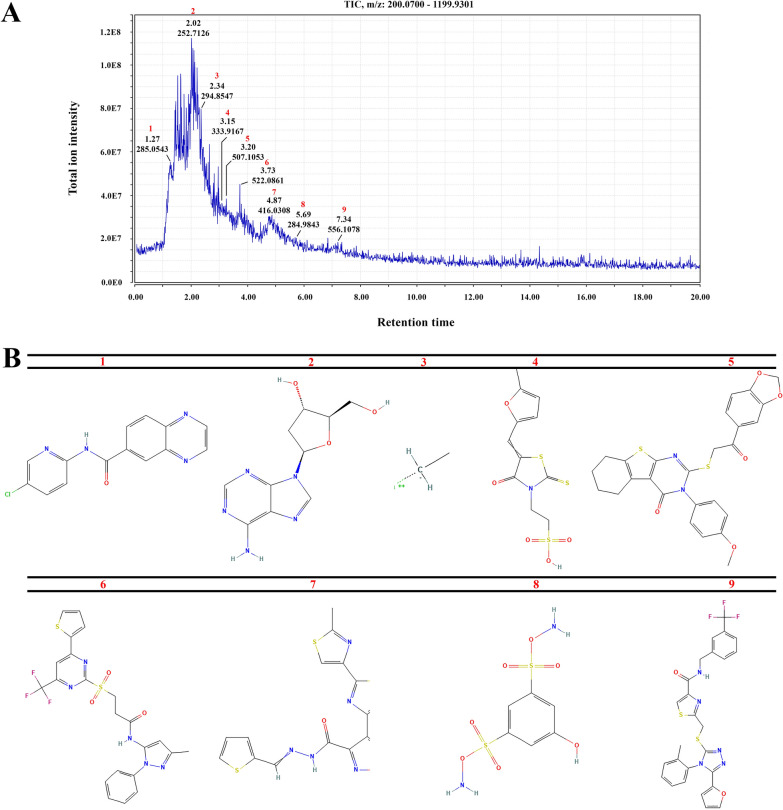
Total ion chromatogram of LC–MS of EtOAc extract in positive ion mode (**A**). Chemical structures of isolated metabolites produced by *Streptomyces* sp. 801 (**B**)

The total ion chromatogram of EtOAc extract obtained from LC–MS analysis showed 9 peaks relating to the following metabolites with potential anti-cancer effects (Fig. 2): *N*-(5-chloropyridin-2-yl) quinoxaline-6-carboxamide (**compound 1**), 2′-Deoxyadenosine (**compound 2**), Barium(2+);ethane;iodide (**compound 3**), 2-[5-[(5-Methylfuran-2-yl)methylidene]-4-oxo-2-sulfanylidene-1,3-thiazolidin-3-yl]ethanesulfonic acid (**compound 4**), 2-{[2-(1,3-benzodioxol-5-yl)-2-oxoethyl]sulfanyl}-3-(4-methoxyphenyl)-5,6,7,8-tetrahydro[1]benzothieno[2,3-d] pyrimidin-4(3 H)-one (**compound 5**), N-(3-methyl-1-phenyl-1 H-pyrazol-5-yl)-3-{[4-(thiophen-2-yl)-6-(trifluoromethyl)pyrimidin-2-yl]sulfonyl}propanamide (**compound 6**), 5-methyl-4-[2-(2-methyl-1,3-thiazol-4-yl)-1,3-thiazol-4-yl]-N-(thiophen-2-ylmethylideneamino)-1,2-oxazole-3-carboxamide (**compound 7**), Diamino 5-hydroxybenzene-1,3-disulfonate (**compound 8**) and 2-[[5-(furan-2-yl)-4-(2-methylphenyl)-1,2,4-triazol-3-yl]sulfanylmethyl]-*N*-[[3-(trifluoromethyl) phenyl]methyl]-1,3-thiazole-4-carboxamide (**compound 9**) (Table 2).

**Table 2:** Chemical compounds isolated from *streptomyces* 801 sp. using LC–MS method

No.	Rt (min)	Formula	[M-H]^+^ (m/z)	Molecular weight	Peak intensity	Pubchem ID	Compund
1	1.27	C_14_H_9_ClN_4_O	285.0543	284.7	5.50E7	829897	*N*-(5-chloropyridin-2-yl) quinoxaline-6-carboxamide
2	2.02	C_10_H_13_N_5_O_3_	252.7126	251.24	1.10E8	13730	2-Deoxyadenosine
3	2.34	C_2_H_5_BaI	294.8547	293.29	6.60E7	12415408	Barium(2+);ethane;iodide
4	3.15	C_11_H_11_NO_5_S_3_	333.9167	333.4	3.50E7	4514835	2-[5-[(5-Methylfuran-2-yl)methylidene]-4-oxo-2-sulfanylidene-1,3-thiazolidin-3-yl]ethanesulfonic acid
5	3.29	C_26_H_22_N_2_O_5_S_2_	507.1053	506.6	3.30E7	1272952	2-{[2-(1,3-benzodioxol-5-yl)-2-oxoethyl]sulfanyl}-3-(4-methoxyphenyl)-5,6,7,8-tetrahydro[1]benzothieno[2,3-d]pyrimidin-4(3 H)-one
6	3.73	C_22_H_18_F_3_N_5_O_3_S_2_	522.0861	521.5	4.50E7	4231522	*N*-(3-methyl-1-phenyl-1 H-pyrazol-5-yl)-3-{[4-(thiophen-2-yl)-6-(trifluoromethyl)pyrimidin-2-yl]sulfonyl}propanamide
7	4.87	C_17_H_13_N_5_O_2_S_3_	416.0308	415.9	4.30E6	2744897	5-methyl-4-[2-(2-methyl-1,3-thiazol-4-yl)-1,3-thiazol-4-yl]-*N*-(thiophen-2-ylmethylideneamino)-1,2-oxazole-3-carboxamide
8	5.69	C_6_H_8_N_2_O_7_S_2_	284.9843	284.3	2.10E7	6365792	Diamino 5-hydroxybenzene-1,3-disulfonate
9	7.34	C_26_H_20_F_3_N_5_O_2_S_2_	556.1078	555.6	1.90E7	1200113	2-[[5-(furan-2-yl)-4-(2-methylphenyl)-1,2,4-triazol-3-yl]sulfanylmethyl]-*N*-[[3-(trifluoromethyl)phenyl]methyl]-1,3-thiazole-4-carboxamide

### Inhibitory effect of the EtOAc extract against cancer cells

**Fig. 3:**
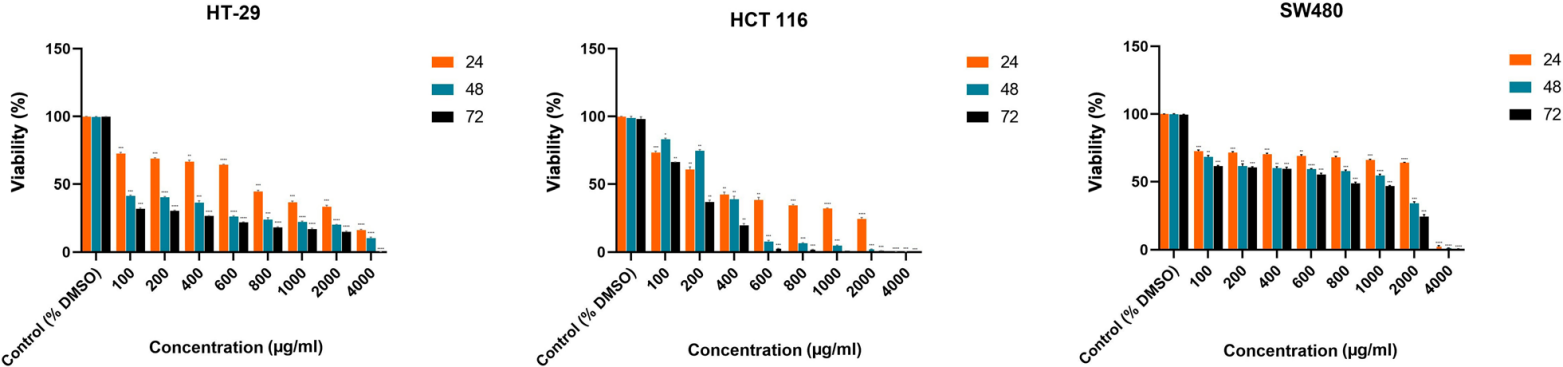
Cytotoxic effect of EtOAc extract on SW480, HCT 116 and HT-29 cell lines. Each cell line was treated for 24, 48 and 72 h, and IC_50_ value was evaluated by MTT assay. All data are shown as mean ± SEM. (*p < 0.05; **p < 0.01; ***p < 0.001; ****p < 0.0001, compared with control)

Figure 3 depicts the results of the MTT assay. In all three cell lines, uninterrupted proliferation was recorded from the control wells. These wells became confluent after 48 h, and their proliferation remained constant. As could be expected, the more aggressive cell lines, SW480 and HCT 116 showed a higher growth rate than HT-29, which is a low-grade colorectal cancer cell line. Between the two more aggressive cell lines, SW480 has metastatic characteristics, while HCT 116 is known as a fast-growing cell line.

The human cells were treated with different concentrations, 100, 200, 400, 600, 800, 1000, 2000, 4000 µg/mL of EtOAc extract for 24, 48 and 72 h. A concentration-dependent decrease in cell viability was observed in all three cell lines. As shown in Fig. 3, after 48 h of incubation, based on the IC_50_ value, the most sensitive cell line to the cytotoxicity of EtOAc extract was identified. The results revealed that the IC_50_ of EtOAc extract on SW480, HCT 116 and HT-29 colorectal cell lines were 861.1, 293.6 and 29.8 µg/mL, respectively.

### Apoptotic effect of EtOAc extract against cancer cells

**Fig. 4:**
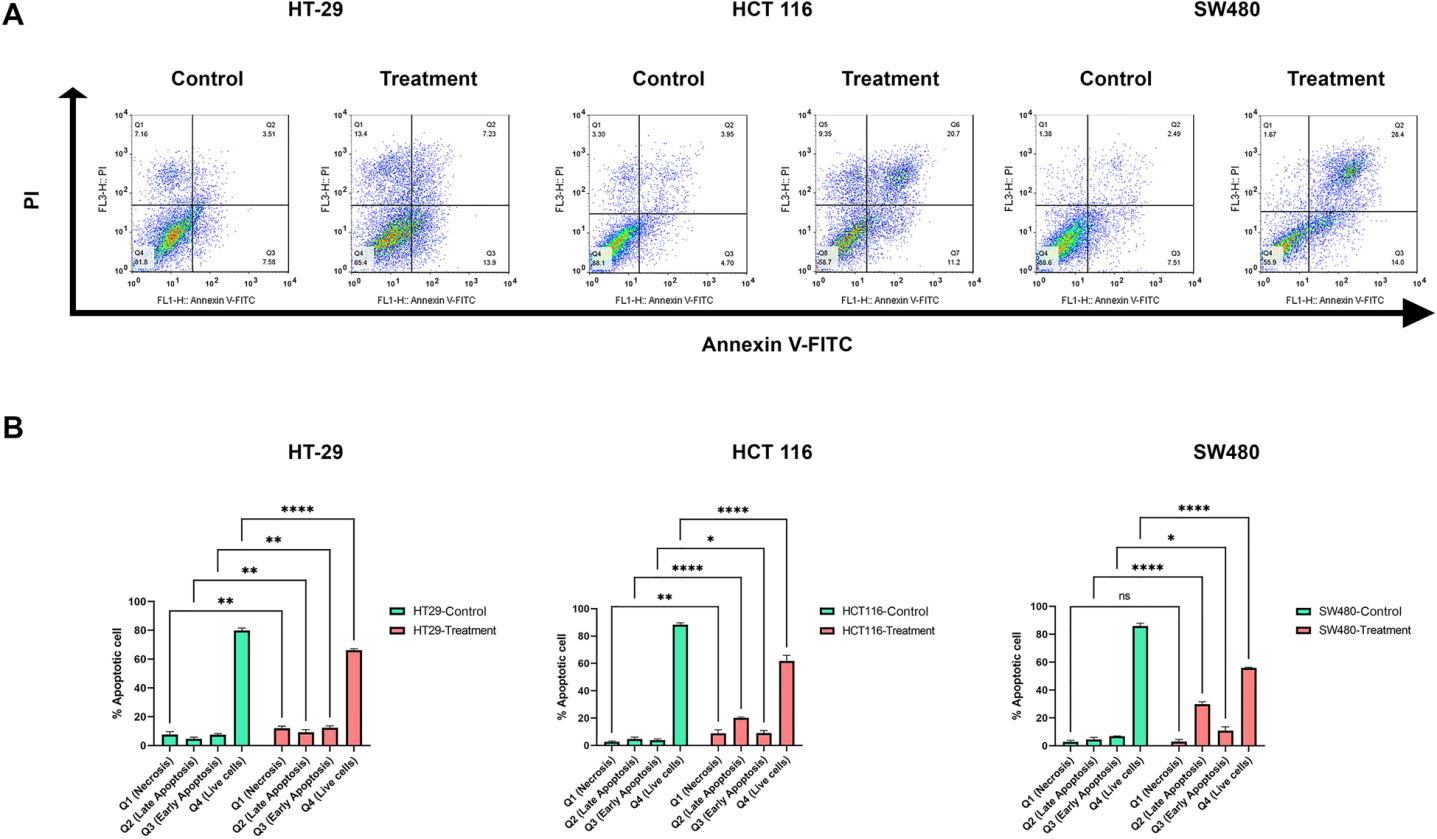
EtOAc extract induced apoptosis in SW480, HCT 116 and HT-29 cell lines. Each cell line was treated with its related IC_50_ value for 48 h, and cell apoptosis was evaluated by Annexin V/PI staining (**A**). Obtained results were quantitated as a chart and data are mean ± SEM (n = 3). *p < 0.05; **p < 0.01; ***p < 0.001; ****p < 0.0001; ns, non-significant compared with control groups (**B**)

Compared to the HT-29, higher percentages of live cells were observed in SW480 and HCT 116 cell lines (Fig. 4). In fact, more aggressive cell lines are probably more resistant and they had naturally fewer apoptotic and necrotic cells than HT-29 cell lines. Annexin V/PI staining showed that the percentage of early and late apoptotic cells was significantly increased from 7.58 to 13.9% and 3.51–7.23% in HT-29 cells (*p < 0.0001*), from 4.70 to 11.2% and 3.95–20.7% in HCT 116 (*p < 0.0001*), and from 7.51 to 14% and 2.49–28.4% in SW480 (*p < 0.0001*), in control compared to the treatment group respectively (Fig. 4).

### Cell cycle analysis of cancer cells treated with the EtOAc extract

**Fig. 5:**
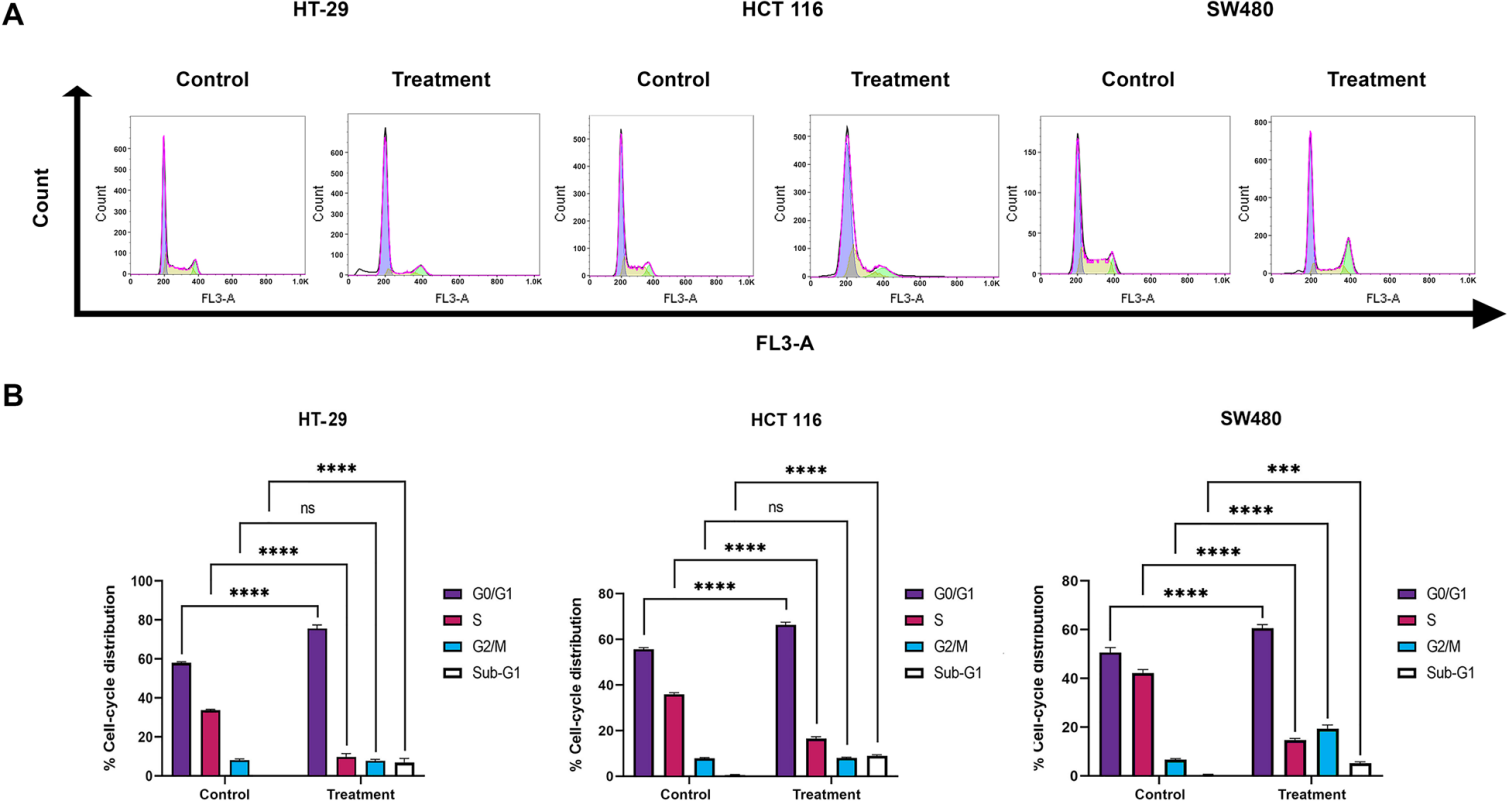
EtOAc extract induced cell cycle arrest in SW480, HCT 116 and HT-29 cell lines. Each cell line was treated with its related IC_50_ value for 48 h, and cell apoptosis was evaluated by flow cytometry (**A**). *p < 0.05; **p < 0.01; ***p < 0.001; ****p < 0.0001; ns, non-significant compared with control groups (**B**)

As more aggressive cell line which are highly active in proleferation and replication, the trasition rate from the G0/G1 phase to the S phase in SW480 and HCT 116 was higher than that of the HT-29 cell line (Fig. 5). Consequently, lower percentage of SW480 and HCT 116 cell lines entered G2/M phase than HT-29 cell line due to staying in S phase.

A significant increase in G0/G1 phase was observed in all three cell lines studied which means hindrance of cell cycle progression into the S phase. Also, the percentage of accumulated cells in the S phase was significantly decreased in HT-29 (from 35% in the control group to 10% in the treatment group), HCT 116 (from 37% in the control group to 17% in the treatment group), and SW480 (from 42% in the control group to 15% in the treatment group). The number of cells in the G2/M phase also increased in SW480 treated cells (19%) compared to the control group (5%) (Fig. 5). These results suggested that *Streptomyces* sp. 801 EtOAc extract hampers cell cycle progression by arresting the cells in the G1 phase and G2/M, afterward leading to inhibition of cell proliferation.

### qRT–PCR analysis of the genes involved in apoptosis and cell cycle

**Fig. 6:**
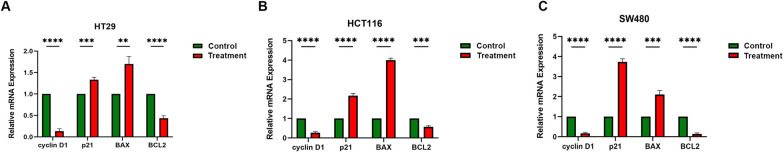
*Bax*, *Bcl-2*, *P21*, and *CCND1* mRNA were assessed by quantitative RT-PCR. Each cell line was treated with its related IC_50_ value for 48 h, and gene expression was evaluated at mRNA level. The qRT-PCR results were normalized against the internal control *GAPDH* and are expressed as a percentage of control cells. Values are the average of 3 separate determinations and shown as mean ± SEM. (*p < 0.05; **p < 0.01; ***p < 0.001; ****p < 0.0001 compared with control)

The relative expression of *Bax*, *Bcl-2*, *CCND1*, and *P21* genes that are involved in cancer was evaluated in this study (Fig. 6). The results showed a significant increase in *Bax* level and a considerable decrease in *Bcl-2* expression level in treated cells compared to the control group, in all three cell lines. Interestingly, the expression of the cell cycle related gene, *Cyclin D1*, decreased, while, *P21* expression was significantly increased (*p < 0.05*) in all cell lines and apoptotic genes. These results demonstrated that the treatment by EtOAc extracts down-regulated important cell cycle regulators and apoptosis genes in colorectal cancer cells.

### The effect of EtOAc extract on cancer cells invasion

**Fig. 7:**
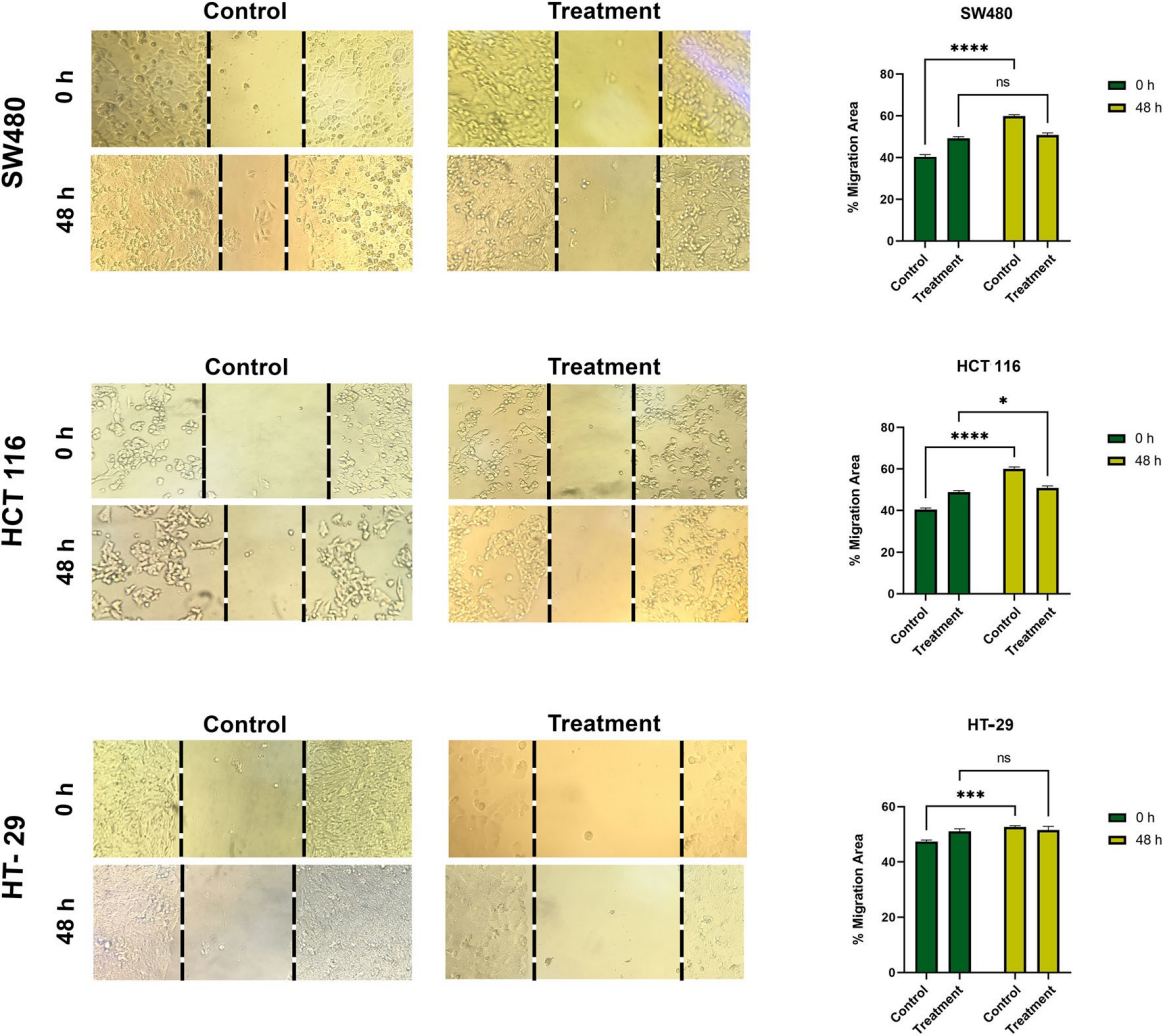
Inhibitory effect of EtOAc extract on migration of SW480, HCT 116 and HT-29 cell lines in a monolayer wound healing model. Phase micrographs of cells were taken at 0 and 48 h after monolayer wounding. Data were quantitated as shown in graph. (*p < 0.05; **p < 0.01; ***p < 0.001; ****p < 0.0001; ns, non-significant compared with control)

In vitro wound assay results for each cell line’s control group showed that cell invasion was mainly reported from the two high-grade cell lines, SW480 and HCT 116. The assay evaluated cell invasion in cultures containing IC50 values for each cell line and vehicle control (Fig. 7). After 48 h of treatment with EtOAc extract, the control group cells were well dispersed in the wound area, while fewer cells were migrated forward in the treated groups in all three cell lines.

## Discussion

Recent research has been focused on the discovery of novel natural anti-cancer metabolites from Actinomycetes because they are impressive sources of bioactive compounds which can be found in the soil and marine environments [39]. Among approximately 33,500 known bioactive metabolites isolated from bacteria, more than 10,400 were reported from the Actinomycetes genus *Streptomyces* [40]. Numerous studies have indicated novel properties of Actinomycetes’ secondary metabolites as antibiotics, enzyme inhibitors, anti-tumor agents, and immunosuppressive chemotherapeutics [41, 42]. Hong et al. (2009) found that 20% of the bacteria isolated from mangrove sites were active against HCT 116 cells, and that the *Streptomyces* showed the highest activity. So far, some anti-cancer drugs have been developed from *Streptomyces* [43]. Adriamycin, isolated from *Streptomyces peucetius*, inhibits DNA replication. Other effective products for cancer chemotherapeutics are actinomycin D, bleomycin, daunorubicin, and mitomycin C. These drugs were obtained from *S. verticillus*, *S. peucetius*, *S. caespitosus*, and other species [44].

In this study, among 19 *Streptomyces* strains of Actinomycetes isolated from soil, *Streptomyces* sp. 801 were found to have an anti-cancer effect against colon cancer cells. Bioactive compounds were extracted by EtOAc and indicated by the LC–MS technique. Various chemical compounds of the organic extract were identified in LC–MS chromatogram, including compounds 1–9.

In a previous study, 47 Actinomycetes were isolated from sediments and seawater samples and evaluated on the resistant MDA-MB-231 breast cancer cell line. All crude extracts facilitated caspase-8 and -10 activation and induced apoptosis in cancer cells mediated by the activation of the endoplasmic reticulum (ER)-stress sensor binding protein (BiP) [45]. Arai et al. reported that Migracin A and B compounds, isolated from the culture filtrate of *Streptomyces* sp., inhibited cell migration of MDA-MB-231, A549, and fibrosarcoma HT1080 cells, without showing any cytotoxicity [46]. Furthermore, they found that migracin A decreased insulin-like growth factor 1 gene (*IGF-1*) expression in ES-2 and JHOC-5 ovarian carcinoma cells. Migracin A also decreased Akt phosphorylation involved in the downstream signaling and inhibited capillary tube formation of human umbilical vein endothelial cells [47]. Ketomycin isolated from a culture filtrate of Actinomycetes SF2912 showed an inhibitory effect on migration and invasion of MDA-MB-231 and MCF-7 breast cancer cells. Ketomycin also decreased the expressions of matrix metallopeptidase genes *MMP*9 and *MMP11* in MDA-MB-231 cells. This antibiotic inhibited the nuclear factor kappa (NF-κB) activity and autophosphorylation of IKK-α/IKK-β as an upstream signaling factor [48]. By Suppressing the metastatic capacities CRC cells, may control tumor development [49]. Here, the wound healing scratch assay showed secondary metabolites derived from the EtOAc extract had an inhibitory effect on migration and invasiveness of all three CRC cell lines.

Extraction and purification of *Streptomyces bingchenggensis* secondary metabolites led to identifying two anti-cancer compounds, ULDF4 and ULDF5. These secondary metabolites were cytotoxic against K562 human acute myelocytic leukemia cells, HeLa cervical carcinoma cells, AGS human gastric cell line, MCF-7, and HL-60 acute promyelocytic leukemia cell lines [25]. Law et al. reported the cytotoxic activity of crude extracts isolated from the 88 *Streptomyces* strain, examined at 400 µg/mL concentration against the HCT-116, HT-29, Caco-2, and SW480 cell lines [50]. Only two of these reported strains, only two strains had more than 50% mortality and the remaining strains had more than 80% of cell viability at this concentration.

In contrast, we found that the organic extracts of *Streptomyces* sp. 801 had cytotoxic activity against SW480, HCT 116, and HT-29 colorectal cancer cells with IC50 values (half-maximal inhibitory concentration) of 861.1, 293.6, and 29.8 µg/mL respectively. Therefore, compared to previously-studied *Streptomyces* species, EtOAc extract can induce significant effects at lower concentrations.

In a recent study, *Streptomyces* sp. MUM256 crude extract isolated from mangrove soil resource produced bioactive metabolites that inhibited the proliferation and induced cell cycle arrest of HCT 116 cells. MUM256 regulated the gene expression level of important cell-cycle regulatory proteins CDK2, cdc25A, p53, Bax, and active caspases-3, -7 and − 9 towards apoptosis induction [51].

*Streptomyces scabrisporus* isolated from soil exhibited significant cytotoxic effect and anti-proliferative activity against N2a mouse neuroblastoma cells, MCF-7, pancreatic cancer MiaPaca-2, prostate cancer PC-3, HCT-116, MDA-MB-231, HL-60 leukemia, and A549 cells. Isolated Alborixin displayed the maximum cytotoxic activity against HCT 116 cells and decreased the clonogenic potential of cancer cells in a dose-dependent manner. Elevating the intracellular ROS level was accompanied by mitochondrial membrane potential loss, reducing the gene expression profile related to the anti-apoptotic protein Bcl-2, increasing cleavage of caspase-3 and PARP-1, activating caspase-8 and -9, and increasing gene expression of pro-apoptotic protein Bax [27].

Our flow cytometry results showed that EtOAc extract could induce apoptosis and cell cycle arrest in all three SW480, HT-29, and HT 116 colorectal cancer cells. In this study, the mRNA level of genes involved in apoptosis and cell cycle, *Bax*, *Bcl-2*, *P21*, and *Cyclin D1*, were investigated using the qRT-PCR method. The critical hallmark features for cancer initiation and progression are primarily assigned to the defect in the apoptotic signaling pathways, undergoing uncontrolled proliferation, development and progression of cancer, and cancer resistance to drug therapies [52]. We found that the mRNA levels of *Bax* and *P21* were increased after treatment of SW480, HT-29, and HT 116 cells by EtOAc extract. The apoptotic mitochondrial events are tightly regulated by the *Bcl-2* family of proteins. The *Bax*, a pro-apoptotic member of the Bcl-2 family, results in permeabilization of the mitochondrial outer membrane [53] and causes cytochrome c from mitochondria to the activation of the caspase cascade [54]. In cancer, pro-apoptotic factors such as *Bax* are suppressed, and anti-apoptotic proteins, such as *Bcl-2*, are upregulated, promoting uncontrolled cell division [55]. Thus, apoptosis induction in cancer cells by modulating the pro-apoptotic proteins involved in the apoptotic pathways is an effective strategy for cancer treatment.

Mutations in cyclin-dependent kinase (CDK)s probably occur during CRC development and progression [56]. Induction of p53 upregulates the *P21* or cyclin-dependent kinases inhibitor 1 A (CDKN1A), P21 inhibits the activity of *cyclin D1* (CCND1) [57], and this finally controls cell cycle regulation, cellular senescence, and stem cell aging in colon cancer cells [58]. Moreover, *cyclin D1* was implicated in adenomatous polyposis coli (APC) signaling: mutated APC cells activate downstream targets, including cyclin D1 [59]. *Cyclin D1* and other CDKs that block cyclins, such as P27 and P21, are vital during the transition from G1 to S phase [59]. In normal colonic mucosa, adenoma, and adenocarcinoma in colon tumors, *Cyclin D1* is highly expressed. This observation indicates that the expression of *Cyclin D1* during the early stages of CRC may deregulate cell cycle control in benign adenomas and stimulate tumor progression [60]. Our results showed that *Bcl-2* and *Cyclin D1*’s expression levels were remarkably decreased in all three colorectal cancer cell lines. Analysis of apoptosis by Annexin V/PI indicated a similar trend of increase in *Bax* and a reduction of *Bcl-2* as observed by qRT-PCR method. Consistent with qRT-PCR results subsequent to the the flow cytometry assay, cell cycle progression was arrested.

Compound 2, 2′-deoxyadenosine, which was derived from the organic phase of *Streptomyces* sp. 801, is a deoxyribonucleoside first reported as an anti-cancer agent against leukemia [61].

Previous studies have shown that the metabolite has more antialgal properties than its analogs, guanosine, 2′-deoxyguanosine, and adenosine. It is also capable of causing irreversible damage to cells before its degradation which results in delayed antialgal activity. Such effects are observed even after “metabolite’s decomposition” [62]. Since the algae is a eukaryote and 2′-deoxyadenosine has shown anti-cancer potential in this study, the property mentioned above can have a long-term effect, which is a notable feature for an effective anti-cancer source.

The present study is the first study that showed the anti-cancer activity of EtOAc extract of a *Streptomyces*, containing 2′-deoxyadenosine with the greatest m/z value among compounds.

Contrary to the previous studies, which pointed out the anti-cancer properties of the synthetic form of this compound [63], this research focused on investigating the metabolites obtained from a natural source. In addition, there is no evidence of an anti-cancer effect of 2′-deoxyadenosine metabolite on colorectal cancer. Purification and isolation of the extracted metabolites using the HPLC method would consolidate the study’s hypotheses. Subsequently, techniques such as Western blotting can be used to evaluate the potential anti-cancer properties of the isolated metabolites [51].

## Conclusions

In conclusion, future studies should be performed to extract and validate the potential compounds of *Streptomyces* strain 801, especially 2′-deoxyadenosine, and elucidate its mode of action. Nonetheless, this study is the first report to shed light on this strain as a promising source for bioactive compounds with anti-cancer activities.

## Current limitations and future perspectives

This study reports the initial steps to introduce a bacterial strain capable of producing bioactive compounds with therapeutic significance. The next steps would be to isolate the candidate compounds and re-examine their effects individually and in combination.

Although many recent studies have been performed on normal and cancer cells, the study is on preliminary investigation of the anti-cancer features of the EtOAc extract against colorectal cancer cells [50, 64, 65].

In addition, AO/PI staining is an optimal method for measuring cell viability for dual-fluorescence detection (live and dead) and eliminating the inaccurate counting of cell populations like cellular debris, which is suggested as a supplementary test in the next steps. All aspects and additional tests such as caspase 3 and 8 will be examined in further studies.

## Data Availability

The datasets generated and/or analysed during the current study are not publicly available due [REASON WHY DATA ARE NOT PUBLIC] but are available from the corresponding author on reasonable request.

## Abbreviations

CRC: Colorectal cancer
GI: Gastrointestinal
EtOAc: Ethyl acetate
LC–MS: Liquid chromatography–mass spectrometry
BiP: Binding protein
CDK: Cyclin-dependent kinase
